# Open channel block of Kv1.5 channels by HMQ1611

**DOI:** 10.3389/fphar.2022.965086

**Published:** 2022-09-16

**Authors:** Chao Dong, Jiawei Li, Weiguang Ding, Rika Ueda, Xiaolu Xie, Jie Wu, Hiroshi Matsuura, Minoru Horie

**Affiliations:** ^1^ Department of Pharmacology, School of Basic Medical Science, Xi’an Jiaotong University Health Science Center, Xi’an, Shaanxi, China; ^2^ Key Laboratory of Environment and Genes Related to Diseases, Xi’an Jiaotong University, Ministry of Education, Xi’an, Shaanxi, China; ^3^ Department of Pharmacy, The First Affiliated Hospital of Xi’an Medical University, Xi’an, China; ^4^ Department of Physiology, Shiga University of Medical Science, Otsu, Shiga, Japan; ^5^ Department of Cardiovascular Medicine, First Affiliated Hospital of Xi’an Jiaotong University, Xi’an, Shaanxi, China; ^6^ Department of Cardiovascular and Respiratory Medicine, Shiga University of Medical Science, Otsu, Shiga, Japan

**Keywords:** HMQ1611, Kv1.5, patch-clamp, molecular docking, open channel block

## Abstract

Kv1.5 channels conduct the ultra-rapid delayed rectifier potassium current (*I*_Kur_). Pharmacological blockade of human Kv1.5 (hKv1.5) has been regarded as an effective treatment of re-entrant based atrial fibrillation, because Kv1.5 is highly expressed in human cardiac atria but scarcely in ventricles. The Kv1.5 blockade is also expected to be used in cancer therapeutics since Kv1.5 is overexpressed in some types of human tumors. Here, we investigated the blockade of hKv1.5 channels by HMQ1611, a symmetrical biphenyl derivative. hKv1.5 channels were heterologously expressed in Chinese hamster ovary cells. The effects of HMQ1611 on wild-type and 13 hKv1.5 mutant channels were examined using the whole-cell patch-clamp method, and molecular docking simulation was conducted to predict the docking position of HMQ1611 within Kv1.5 channels. We showed that HMQ1611 reversibly inhibited the hKv1.5 current in a concentration-dependent manner (IC_50_ = 2.07 μM). HMQ1611 blockade of hKv1.5 current developed with time during depolarizing voltage-clamp steps, and this blockade was also voltage-dependent with a steep increase over the voltage range for channel openings. HMQ1611 inhibition was significantly reduced in the T479A, T480A, V505A, I508A, L510A, V512A, and V516A hKv1.5 mutant channels. Molecular docking analysis predicted that V505, V512, and T480 were involved in the blocking action of HMQ1611 on hKv1.5 channels. These results suggest that HMQ1611 inhibits hKv1.5 currents as an open channel blocker. Amino acid residues located at the base of the selectivity filter (T479 and T480) and in the S6 segment (V505, I508, L510, V512, and V516) of hKv1.5 appear to constitute potential binding sites for HMQ1611.

## Introduction

Kv1.5 channel, encoded by KCNA5 gene, is the main molecular component of the ultra-rapid delayed rectifier potassium ion channel (*I*_Kur_) (Fedida et al., 1993; Chiamvimonvat et al., 2017). This channel belongs to the Shaker-type (Kv1) voltage-gated potassium channel family and comprises four pore-forming subunits, each containing six transmembrane segments (S1–S6) (Ravens and Odening, 2017). The Kv1.5 channel current is one of three repolarizing K^+^ currents (*I*_Kur_, *I*_Kr,_ and *I*_Ks_) during the plateau and repolarization phases of the cardiac action potential (AP). As the Kv1.5 channel is expressed in the atria but scarcely in the ventricles of the human heart and the functional current of the channel is only detected in the atria (Ravens and Odening, 2017; Borrego et al., 2021), blockade of this channel can be expected to exert antiarrhythmic actions against re-entrant based atrial fibrillation (AF) without causing ventricular arrhythmias (e.g., torsade de pointes, TdP) (Wijesurendra and Casadei, 2019; Heijman et al., 2021). Moreover, several recent studies have revealed that the Kv1.5 channel is aberrantly expressed in numerous human cancers and plays crucial roles in cancer development (Comes et al., 2013; Comes et al., 2015; Ravens and Odening, 2017), suggesting that the Kv1.5 channel might be a potential molecular target for anticancer therapies.

In the past decade, considerable efforts have been made to develop blockers of the Kv1.5 channel (Voigt and Dobrev, 2016; Ravens and Odening, 2017; Attali et al., 2019; Borrego et al., 2021) and to explore the molecular binding sites involved in the blocking action of some Kv1.5 blockers (Wu et al., 2009; Bai et al., 2015; Wu and Sanguinetti, 2016; Chen and Chung, 2018; Fukushima et al., 2020). A few known Kv1.5 channel blockers (e.g., BMS-394136, BMS-919343, MK-0448, F373280, XEN-D0101, and XEND0103/S66913) have entered clinical development for AF (EI-Haou et al., 2015). However, to date, only Vernakalant (BRINAVESS) has been introduced into clinical therapeutics for AF in Europe (Brown et al., 2014). As Vernakalant can also block several other cardiac ion channels, it appears that although *I*_Kur_ blockade may contribute to its antifibrillatory effect, the most important action of this drug on AF is through atrial-selective blockade of peak sodium current (Burashnikov et al., 2012; Chiamvimonvat et al., 2017; Ravens and Odening, 2017).

HMQ1611 is a derivative of symmetrical biphenyl (Zhang et al., 2011) that is shared with LY294002, a phosphoinositide 3-kinase (PI3K) blocker showing Kv1.5 channel blockade and the capacity to increase the AP duration (APD) in isolated mouse myocytes (Sun et al., 2004; Wu et al., 2009). Similar to LY294002 (Ebrahimi et al., 2017), HMQ1611 also exhibits potent anticancer activity (Zhang et al., 2011). It is therefore hypothesized that HMQ1611 has a Kv1.5 channel-blocking ability similar to that of LY294002. The present study sought to explore the inhibitory effect of HMQ1611 on the human Kv1.5 (hKv1.5) channel and the molecular binding modes of HMQ1611 with the hKv1.5 channel, using both the whole-cell patch-clamp technique and an *in silico* docking simulation model.

## Materials and methods

### Cell culture, site-directed mutagenesis, and transfection

Chinese hamster ovary (CHO) cells donated by Dr. Takeru Makiyama (Kyoto University Graduate School of Medicine, Japan) were cultured in Dulbecco’s modified Eagle’s medium/Ham’s F-12 (DMEM/F-12) medium supplemented with 10% fetal bovine serum and antibiotics (100 IU/ml penicillin and 100 μg/ml streptomycin) in a humidified atmosphere with 5% CO_2_ and 95% air at 37°C. Cells were passaged every 3–4 days with trypsin–EDTA, and a portion of the cells was seeded onto glass cover slips (5 × 3 mm^2^) for subsequent transfection.

Full-length cDNA encoding wild-type (WT) hKv1.5 (KCNA5) subcloned into the pcDNA3.1 expression vector was kindly provided by Dr. David Fedida (University of British Columbia, Canada). All hKv1.5 mutants (T462C, H463C, T479A, T480A, R487V, A501V, I502A, V505A, I508A, A509G, L510A, V512A, and V516A cDNAs) were constructed using a QuickChange II XL site-directed mutagenesis kit according to the manufacturer’s instructions (Stratagene, La Jolla, CA, USA) and subcloned into the pcDNA3.1. All mutants were fully sequenced (ABI3100x; Applied Biosystems, Foster City, CA, USA) to ensure fidelity.

WT or mutant hKv1.5 cDNA was transiently transfected into CHO cells together with green fluorescent protein (GFP) cDNA (0.5 μg WT or mutant hKv1.5 + 0.5 μg GFP) using Lipofectamine (Invitrogen Life Technologies, Carlsbad, CA, USA). Patch-clamp experiments were conducted on GFP-positive cells 48 h after transfection.

### Electrophysiological recordings and data analysis

Kv1.5 currents were recorded using an EPC-8 patch-clamp amplifier (HEKA, Lambrecht, Germany) at 25°C. The data were low-pass filtered at 1 kHz, acquired at 5 kHz through an LIH-1600 analogue-to-digital converter (HEKA), and stored on a hard disc drive using Pulse/PulseFit software (HEKA). For experiments to measure the activation time course of hKv1.5 currents, the data were low-pass filtered at 10 kHz and sampled at 50 kHz. Patch electrodes had a resistance of 2.5–4.0 MΩ when filled with the pipette solution. Cells attached to glass cover slips were transferred to a recording chamber (0.5 ml in volume) mounted on the stage of an inverted microscope (ECLIPSE TE2000-U, Nikon, Tokyo, Japan) and perfused continuously with Tyrode solution at a flow rate of 1 ml/min.

hKv1.5 channel currents were elicited by applying 300 ms depolarizing steps from a holding potential of -80 mV to various levels of -50 mV to +50 mV in 10 mV steps with a return potential of -40 mV, and each stimulus interval was longer than 10 s. Voltage-dependent activation of hKv1.5 channels was assessed by fitting the normalized *I*–*V* relations of the tail currents to a Boltzmann equation:

$$I_{\text{tail}}=\frac{1}{\left(1+\exp\left(\left(V_{1/2}-V_{m}\right)/k\right)\right)},$$ (1)

where *I*_tail_ is the tail current amplitude normalized with reference to the maximum value measured at +50 mV, *V*_1/2_ is the voltage at half-maximal activation, *V*_m_ is the test potential, and *k* is the slope factor.

The concentration–response curve for inhibition of hKv1.5 current by HMQ1611 was drawn by a least-squares fit of the Hill equation:

$$\%\text{Control}=\frac{1}{\left(1+{\left(IC_{50}/\left[D\right]\right)}^{nH}\right)},$$ (2)

where % Control represents the current in presence of the drug normalized with reference to the control amplitude (expressed as a percentage), IC_50_ is the concentration of HMQ1611 causing a half-maximal inhibition, n_H_ is the Hill coefficient, and [D] is the drug concentration.

The apparent rate constants for binding (*k*_+1_) and unbinding (*k*_-1_) were obtained by fitting the equation:

$$\tau_{D}=\frac{1}{\left(k_{+1}\left[D\right]+k_{-1}\right)},$$ (3)

where τ_D_ is the drug-induced time constant, which was calculated from single exponential fits to the traces of current decay during the depolarizing step to +30 mV.

The apparent dissociation constant *K*_D_ is expressed as

$$k_{D}=\frac{k_{-1}}{k_{+1}}.$$ (4)

Deactivation kinetics of hKv1.5 were determined by fitting a single exponential function to the tail current trace (obtained by depolarizing a cell to +30 mV for 300 ms, followed by a repolarizing pulse to -40 mV).

### Solutions and chemicals

Normal Tyrode solution contained 140 mM NaCl, 5.4 mM KCl, 1.8 mM CaCl_2_, 0.5 mM MgCl_2_, 0.33 mM NaH_2_PO_4_, 5.5 mM glucose, and 5.0 mM HEPES (pH adjusted to 7.4 with NaOH). The pipette solution contained 70 mM potassium aspartate, 40 mM KCl, 10 mM KH_2_PO_4_, 1 mM MgSO_4_, 3 mM Na_2_-ATP (Sigma Chemical Company, St Louis, MO, USA), 0.1 mM Li_2_-GTP (Sigma), 5 mM EGTA, and 5 mM HEPES (pH adjusted to 7.2 with KOH). HMQ1611 was dissolved in dimethyl sulfoxide (DMSO; Sigma) to yield a 50 mM stock solution. The concentration of DMSO in the final solution was <0.1% (*V*/*V*), which had no effect on the hKv1.5 currents.

### Molecular docking analysis

The modeling of hKv1.5 channels, drug docking, calculation of binding free energy, and representation of docking were integrated using the drug discovery software platform Molecular Operating Environment (MOE) 2016.0802 (Chemical Computing Group, Inc., Quebec, Canada), as described previously (Fukushima et al., 2020). Based on the 2.9 Å crystal structure of Kv1.2 channel (Protein Data Bank accession code: 2A79), which is approximately 90% homologous in amino acid sequence (from Asp207 to Thr527) to the pore domain with the hKv1.5 channel, an open-state homology model of hKv1.5 pore domain was constructed using the MOE software platform (Fukushima et al., 2020). HMQ1611 was docked to the Kv1.5 model using Dock in the MOE program. We adopted the Amber10: EHT force field to set the force-field parameters and then calculated the HMQ1611 binding energy using the Kv1.5 model. During docking simulation, the channel structure remained rigid, whereas HMQ1611 was flexible. The calculated free energy of binding (S score) was used to assess HMQ1611 docking to the channel, and the amino acids within 4.0 Å of the docked compound were identified as having potential contact.

### Statistical analysis

All averaged data are presented as mean ± SEM (standard error of the mean, *n* = number of cells). Student’s *t*-test or analysis of variance (ANOVA) with Dunnett’s post hoc test was used to evaluate statistical significance, which was considered to be statistically significant if a *p* value was <0.05.

## Results

### Inhibitory action of HMQ1611 on hKv1.5 current

**FIGURE 1:**
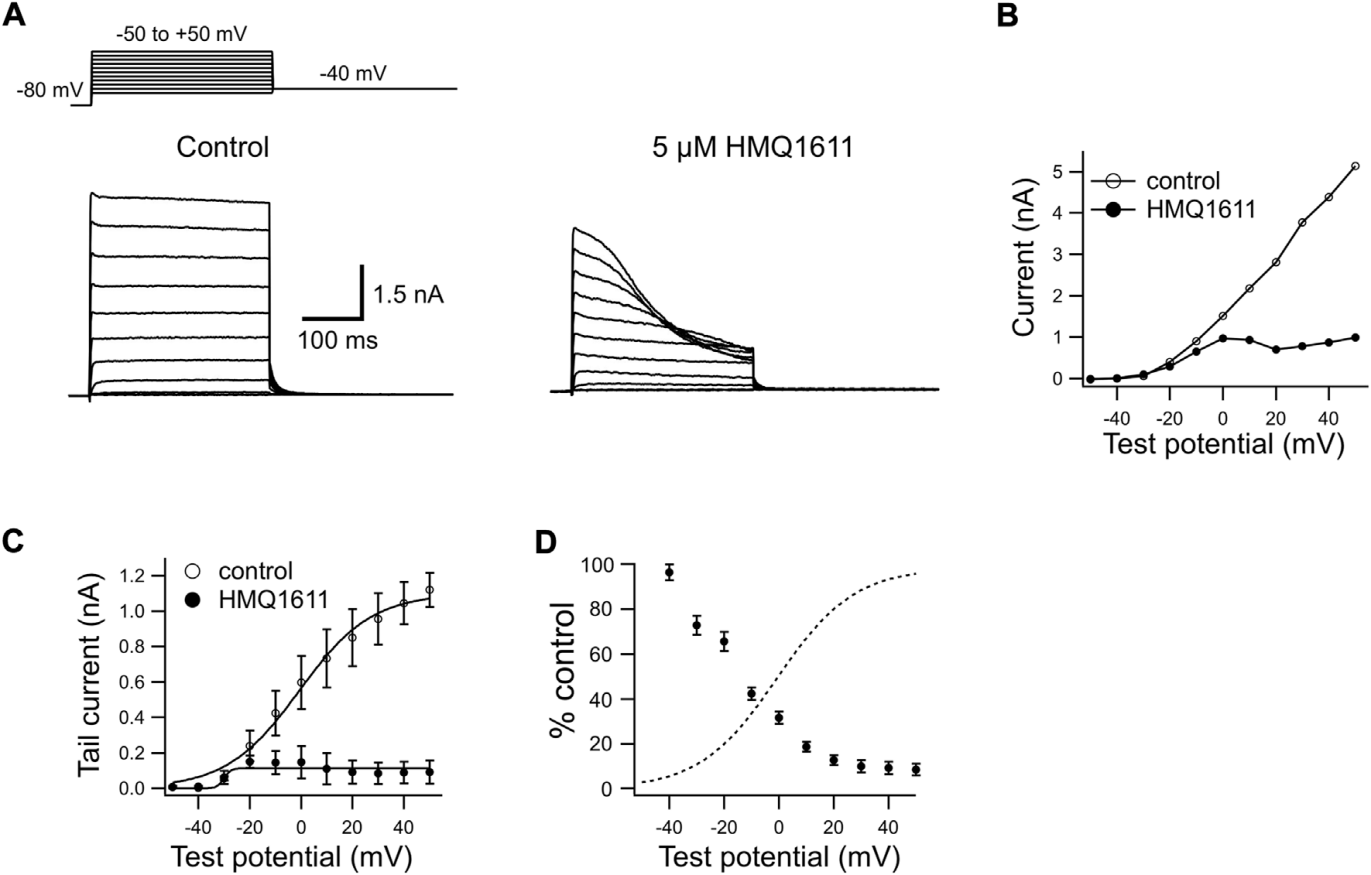
Blocking properties of HMQ1611 on human Kv1.5 (hKv1.5) channels heterologously expressed in Chinese hamster ovary cells. **(A)** Superimposed hKv1.5 channel currents elicited during 300 ms depolarizing steps given from a holding potential of -80 mV to potentials between -50 and +50 mV in 10 mV increments, followed by a 300 ms repolarization step to -40 mV before (Control) and after exposure to 5 μM HMQ1611. **(B)** Current-voltage (*I*–*V*) relations of hKv1.5 currents measured at the end of 300 ms depolarization under control conditions and during exposure to 5 μM HMQ1611. **(C)** *I*–*V* relations for tail currents in control and during exposure to 5 μM HMQ1611. The smooth curves through the data points represent the least-squares fit to a Boltzmann equation. **(D)** Late current amplitude in the presence of HMQ1611 is plotted as a percentage of control amplitude in the absence of the compound (% Control, filled circles). The dashed curve represents the activation curve obtained in control conditions (see Figure 1C).

Figure 1A shows representative current traces recorded in a CHO cell heterologously expressing the hKv1.5 channel. The superimposed hKv1.5 currents were evoked by 300 ms depolarizing voltage-clamp steps given from a holding potential of -80 mV to various test potentials with a return potential of -40 mV (Figure 1A inset), in the absence (control) and in presence of 5 μM HMQ1611. Under control conditions, hKv1.5 currents were rapidly evoked upon depolarization to reach a peak and then remained stable during depolarized test steps, consistent with previous studies (Wu et al., 2009; Bai et al., 2015; Fukushima et al., 2020). The bath application of 5 μM HMQ1611 produced a marked, time-dependent decline in outward currents during each depolarizing test potential. Figure 1B illustrates *I*–*V* relationships for late currents (measured at the end of 300 ms clamp steps) in the absence and presence of HMQ1611. The voltage-dependent activation of hKv1.5 currents was evaluated by fitting a Boltzmann equation to the amplitude of tail currents elicited on return to -40 mV following depolarizing voltage steps to various test potentials (Figure 1C). In a total of 6 cells, *V*_1/2_ averages were -0.76 mV and -29.9 mV (*p* < 0.01 vs*.* control) in control and during exposure to 5 μM HMQ1611 respectively, while *k* were 14.09 and 1.34 (*p* < 0.01 vs*.* control), respectively. Such a negative shift in *V*_1/2_ does not mean that the HMQ1611 affected the channel gating kinetics. HMQ1611 exhibited a stronger blocking effect on the hKv1.5 channel when depolarizing stimuli were more intense, and the blocking effect reached a saturated state at a certain depolarizing potential after the channel open. The characteristics of hKv1.5 blockade by HMQ1611 are shared with a few other compounds (Wu et al., 2009; Bai et al., 2015; Kojima et al., 2015). Woodhull previously reported that hydrogen ions blocked sodium in a voltage-dependent manner in nerve (Woodhull, 1993). Hydrogen ions enter open sodium channels and stick inside the channels, blocking the sodium permeability and shifting the responses of sodium channel gates to voltage while responding to membrane voltage. Likewise, the Woodhull block model is very likely to be suitable for the Kv1.5 channel. The relative amplitude of late currents in the presence and absence of HMQ1611 (% control) was measured at each test potential and plotted (Figure 1D) together with the activation curve (dashed line) obtained in the control condition. The current reduction steeply increased at more positive potentials between -40 mV and 0 mV, which corresponded to the voltage range of the channel opening. At potentials positive to +20 mV, where the channel conductance was nearly saturated, the current reduction exhibited a shallow voltage dependence. These results suggest that HMQ1611-induced inhibition of hKv1.5 currents occurs preferentially after channels open. Meanwhile, HMQ1611 did not appreciably affect the deactivation kinetics of hKv1.5. In the absence and in presence of 5 μM HMQ1611, the deactivation time constants were 14.8 ± 1.4 ms and 13.1 ± 2.3 ms (*n* = 10, *p* > 0.05 vs*.* control), respectively.

### Concentration-dependent inhibition of hKv1.5 current by HMQ1611

**FIGURE 2:**
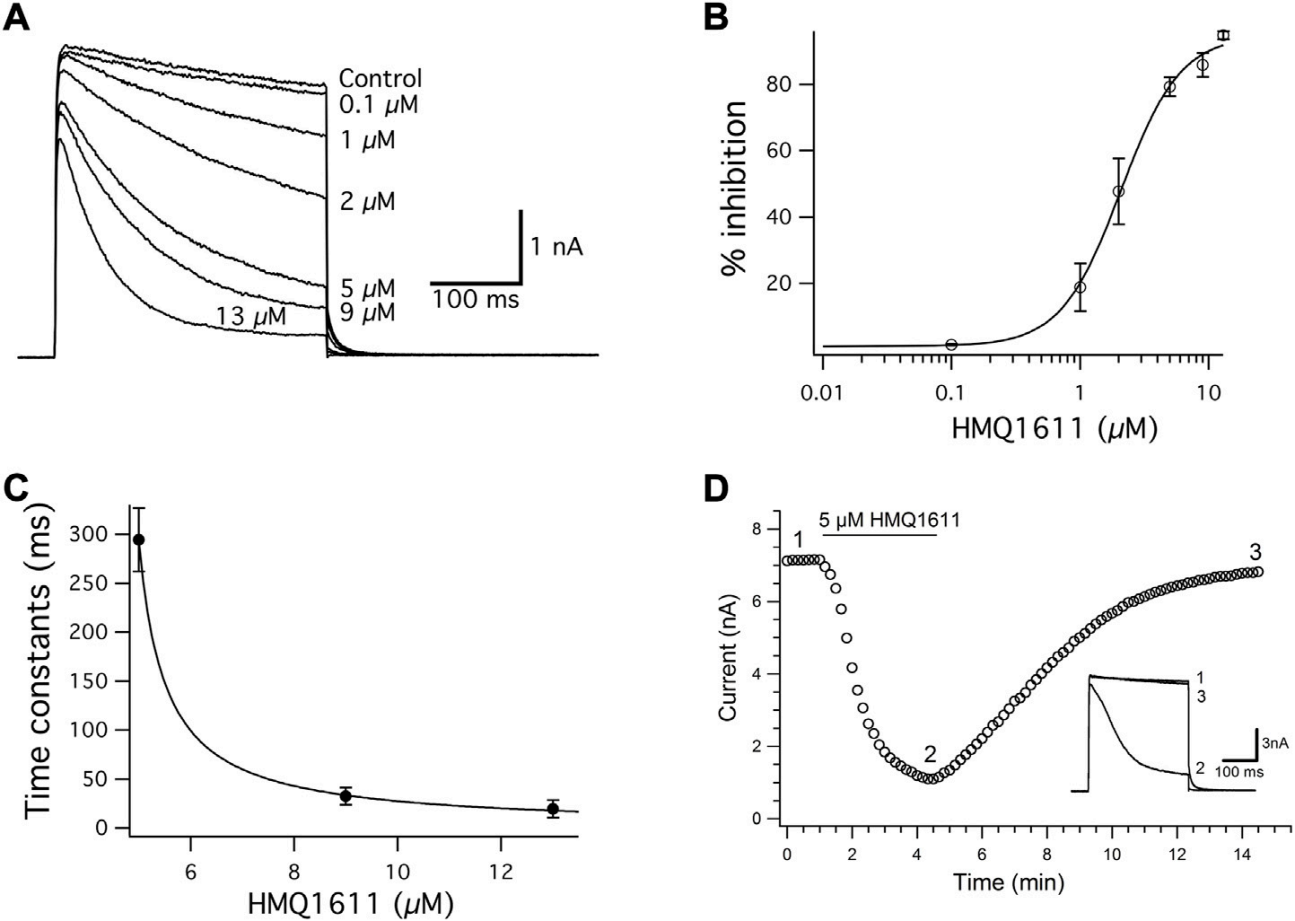
Concentration-dependent block of hKv1.5 current by HMQ1611. **(A)** Superimposed hKv1.5 current traces were elicited by applying 300 ms depolarizing pulses from a holding potential of -80 mV to +30 mV every 10 s in the absence (control) and presence of various concentrations of HMQ1611 (0.1, 1, 2, 5, 9, and 13 μM). **(B)** Concentration–response relationship for the inhibition of hKv1.5 current by HMQ1611. Percentage inhibition (% inhibition) represents the fraction of hKv1.5 current reduced by each concentration of HMQ1611 with reference to the control amplitude, measured at the end of 300 ms depolarizing pulse to +30 mV. The smooth curve through the data points represents a least-squares fit of a Hill equation, yielding an IC_50_ of 2.07 ± 0.21 μM and a Hill coefficient (n_H_) of 1.87 ± 0.32 (*n* = 7). **(C)** Kinetics of HMQ1611 block of hKv1.5 current. The time constant (τ_D_) for tail current during the depolarization potential of +30 mV at various concentrations of HMQ1611 was plotted as a function of the drug concentration (1, 9, and 11 μM). The solid line represents the fit of the data to the hyperbolic function. **(D)** Reversible inhibition of hKv1.5 current by 5 μM HMQ1611. The current amplitude of hKv1.5 were measured at the end of 300 ms depolarizing step from a holding potential of -80 mV to +30 mV every 10 s, before (control), in the presence of 5 μM HMQ1611, and after its washout. Inset shows the current trace, which was recorded at different time points.

Figure 2A illustrates the inhibitory effects of HMQ1611 on hkv1.5 at various concentrations between 0.1 and 13 μM. The hKv1.5 current was elicited every 15 s by a 300 ms depolarizing step to +30 mV, before (control) and during exposure to increasing concentrations of HMQ1611 in a cumulative manner, after the inhibition due to the previous concentration reached a steady state (Figure 2A). It is shown that bath application of HMQ1611 did not significantly affect the activation time constant (control: 1.50 ± 0.25 ms; 5 μM of HMQ1611: 1.47 ± 0.18 ms, *n* = 7, *p* > 0.05 vs*.* control). However, at each given concentration of HMQ1611, the late current level at the end of the 300 ms clamp step was more potently reduced than the initial peak current level, which supports the gradual development of channel inhibition during the open state of the channel.

Figure 2B illustrates the concentration–response relationship for the inhibition of hKv1.5 current by HMQ1611, measured at the end of the depolarizing step to +30 mV in seven different cells. The mean data were well fitted with a Hill equation with IC_50_ of 2.07 ± 0.21 μM and a Hill coefficient of (n_H_) of 1.87 ± 0.32 (*n* = 7). HMQ1611 also caused a concentration-dependent acceleration of hKv1.5 current decay during the depolarizing step (to +30 mV). Figure 2C shows the time constant (τ_D_) of block development during a depolarization potential of +30 mV at three different concentrations of HMQ1611. Data points were fitted to a hyperbolic equation (Eq. 3) to the tail current trace, yielding apparent binding (*k*_+1_) and unbinding (*k*_-1_) rate constants that averaged 6.65/(μM·s) and 29.9/s, respectively. The K_D_ value derived by (*k*_-1_)/(*k*_+1_) was 4.48 μM, which is close to the value of IC_50_ (2.07 μM), suggesting that HMQ1611 may prefer to bind to hKv1.5 channel in an open state.

Figure 2D illustrates a representative time course of the hKv1.5 current measured at the end of the 300 ms depolarizing step to +30 mV in the presence of 5 μM HMQ1611 and after washout with normal Tyrode solution (control). The hKv1.5 current started to get inhibited within 5–10 s on exposure to 5 μM HMQ1611 and reached a steady state level (approximately 86% inhibition) within 3 min. The inhibitory effect on the hKv1.5 current was largely reversed (recovered to 94.64% of the control value) within 10 min after washout with normal Tyrode solution, which indicates that HMQ1611 is a reversible blocker of the hKv1.5 channel.

### Frequency-dependent inhibition of hKv1.5 current by HMQ1611

**FIGURE 3:**
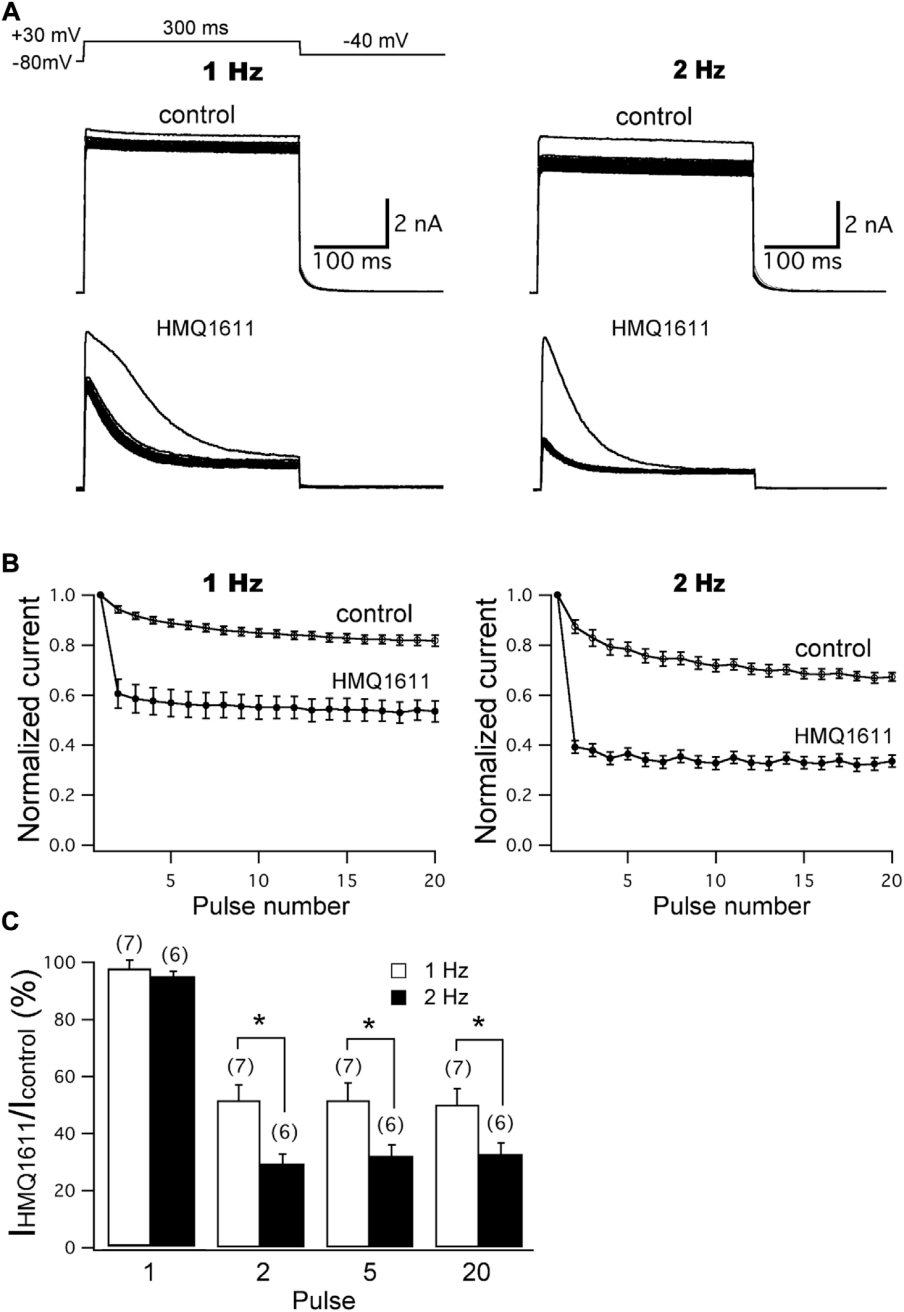
Frequency-dependent block of hKv1.5 current by HMQ1611. **(A)** Original current traces of hKv1.5 in the absence and presence of 5 μM HMQ1611 at two different frequencies (1 and 2 Hz) were obtained using a train of 20 depolarizing steps of 200 ms duration from -80 mV to +50 mV followed by a 200 ms repolarization step to -40 mV. **(B)** Peak amplitudes of outward current at every pulse were normalized with reference to the peak amplitude of current obtained at the first pulse and then plotted against the pulse numbers. **(C)** Normalized current amplitudes for pulse number 1, 2, 5, and 20 before and after the application of 5 μM HMQ1611, obtained at the two frequencies. Experiment numbers are shown in parenthesis. Data are demonstrated as mean ± SEM. ^*^ *p* < 0.05 vs*.* normalized current amplitude at 1 Hz.

The frequency-dependent and use-dependent block of hKv1.5 channels by 5 μM HMQ1611 were examined at two different frequencies (1 and 2 Hz) using a train of 20 depolarizing steps with 300 ms duration from -80 mV to +30 mV. This was followed by a 200 ms repolarization step to -40 mV (Figure 3A inset). The cell was equilibrated for 3 min at -80 mV to keep the channels in the closed state, so that the first sweep represents the first channel opening. Superimposed current traces of hKv1.5 in Figure 3A show that, in the presence of 5 μM HMQ1611, the peak current amplitudes at the first depolarizing step were not significantly (*p* > 0.05) modified at both 1 Hz (control: 6.11 ± 0.99 nA; HMQ1611: 5.97 ± 0.98 nA, *n* = 7) and 2 Hz (control: 5.82 ± 1.14 nA; HMQ1611: 5.67 ± 0.99 nA, *n* = 6), indicating that the compound did not bind to the channel in the rested state. The normalized peak current amplitudes at 1 and 2 Hz before (control) and during exposure to 5 μM HMQ1611 are shown in Figure 3B. Under control conditions, the peak amplitudes of hKv1.5 current at the 20th depolarizing step reduced by 18.29 ± 2.20% (*n* = 7) at 1 Hz and 32.73 ± 1.74% (*n* = 6) at 2 Hz, compared with the first pulse. In the presence of 5 μM HMQ1611, the peak amplitudes of hKv1.5 current progressively decreased by 46.57 ± 4.19% (*n* = 7) and 66.47 ± 2.38% (*n* = 6) at the 20th pulse at 1 Hz and 2 Hz, respectively. Figure 3C shows the normalized current amplitudes (I_HMQ1611_ */*I_Control_) measured for pulse numbers 1, 2, 5, and 20 at 1 Hz and 2 Hz. These normalized current amplitudes were significantly (*p* < 0.05) lower at 2 Hz than at 1 Hz for pulse numbers 2, 5, and 20. Therefore, the degree of inhibition of hKv1.5 increased as the pulse frequency increased, suggesting that HMQ1611 blocks hKv1.5 channels in a frequency-dependent and use-dependent manner.

### Effects of HMQ1611 on hKv1.5 mutant channels

To further explore the putative binding sites of hKv1.5 channel that HMQ1611 acts on, 13 amino acids (T462, H463, T479, T480, A487, A501, I502, V505, I508, A509, L510, V512, and V516) located in the pore and S6 domain of hKv1.5 were mutated by site-directed mutagenesis. Among these amino acids, T462 and H463 were located at the pore turret, T479 and T480 were located at the base of the ion selectivity filter, A487 was located at the external entryway of the pore, and A501, I502, V505, I508, A509, L510, V512, and V516 were located within the S6 segment.

**FIGURE 4:**
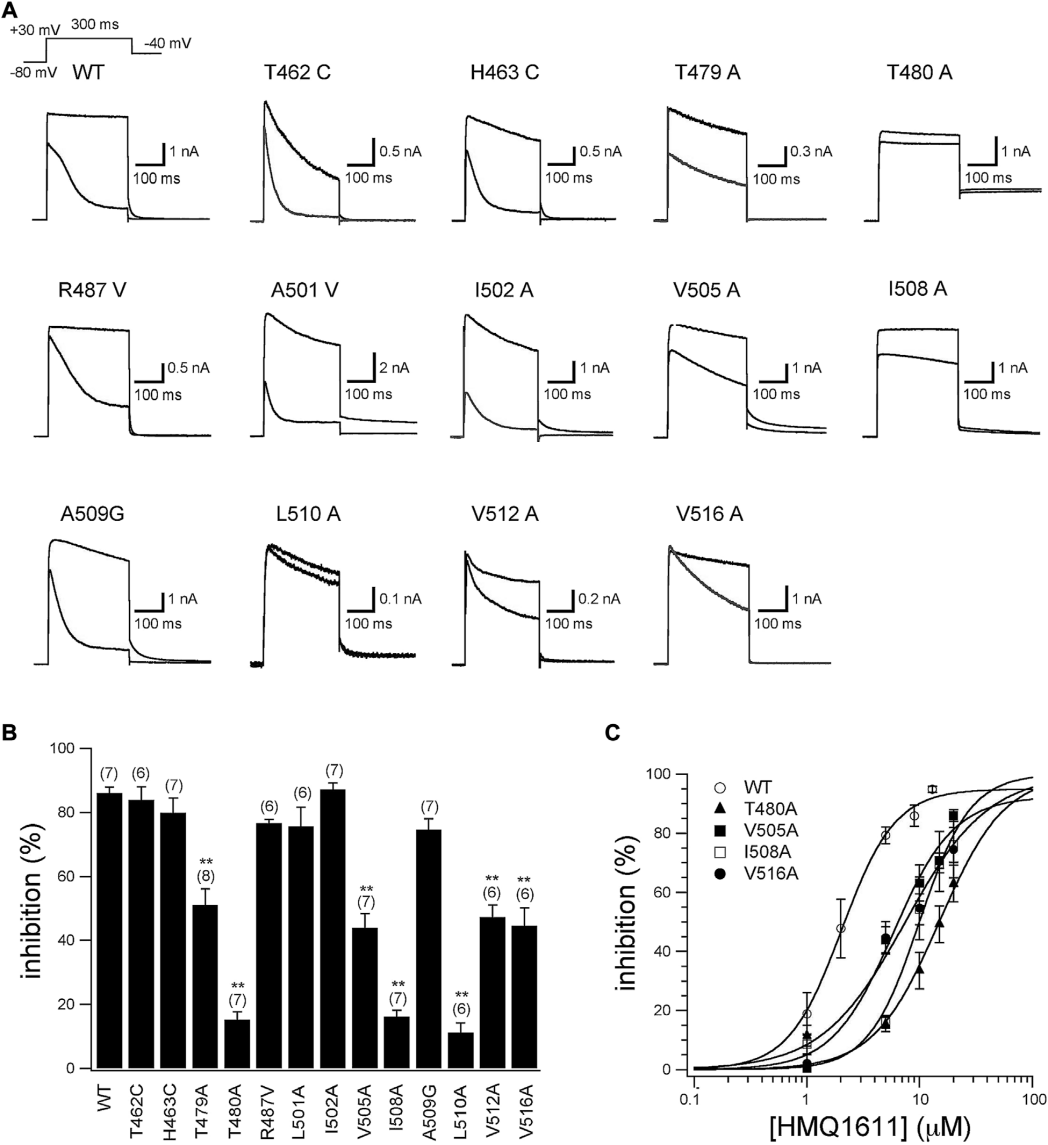
Inhibitory action of HMQ1611 on WT and thirteen hKv1.5 mutant channels. **(A)** Current traces recorded from wild type (WT) and 13 hKv1.5 mutant channels in CHO cells, using a 300 ms depolarizing step to +30 mV from a holding potential of -80 mV in the presence and absence of 5 μM HMQ1611. **(B)** Percentage inhibition of WT and 13 hKv1.5 mutant channels by 5 μM HMQ1611. Currents were measured at the end of 300 ms depolarizing pulse. Experiment numbers are shown in parenthesis. Data are demonstrated as mean ± SEM. ^**^ *p* < 0.01 vs*.* WT. **(C)** Concentration-response relationship curves for HMQ1611 block of WT and hKv1.5 mutant channels (T480A, V505A, I508A, V516A) (six to eight cells in each channel). The solid lines represent a least-squares fit of a Hill equation.

Figure 4A shows the representative current traces of WT and 13 hKv1.5 mutant channels (T462C, H463C, T479A, T480A, R487V, A501V, I502A, V505A, I508A, A509G, L510A, V512A, and V516A) activated with a 300 ms depolarizing step to +30 mV from a holding potential of -80 mV in the presence and absence of 5 μM HMQ1611. As shown in Figure 4B and Table 1, the degree of blockade of late currents by HMQ1611 was significantly attenuated in T479A, T480A, V505A, I508A, L510A, V512A, and V516A mutant hKv1.5 channels. We further explore the concentration-response relationship for the inhibition of four hKv1.5 mutant channels by HMQ1611 (Figure 4C), which yields an IC_50_ of 16.31 ± 2.11 μM (n_H_ = 1.75 ± 0.11, *n* = 7) for T480A mutant, an IC_50_ of 6.44 ± 0.18 μM (n_H_ = 1.55 ± 0.19, *n* = 7) for V505A, an IC_50_ of 10.50 ± 0.41 μM (n_H_ = 1.98 ± 0.07, *n* = 7) for I508A, and an IC_50_ of 8.29 ± 1.2 μM (n_H_ = 1.34 ± 0.23, *n* = 6) for V516A. Compared with IC_50_ of WT channel (2.07 ± 0.21 μM, *n* = 7), those of these four mutant channels exhibit 7-fold, 3-fold, 5-fold, and 4-fold decrease in blocking potency, respectively. These data further indicate that the pore and S6 domain sites of hKv1.5 are important binding targets for the blockade by HMQ1611.

**TABLE 1:** Inhibition of HMQ1611 on mutant hKv1.5 channels.

	WT(*n* = 7)	T479A(*n* = 8)	T480A (*n* = 7)	V505A (*n* = 7)	I508A (*n* = 7)	L510A (*n* = 6)	V512A (*n* = 6)	V516A (*n* = 6)
Inhibition (%)	83.94 ± 4.06	51.11 ± 4.99**	15.32 ± 2.5**	43.95 ± 4.4**	16.18 ± 2.09**	11.16 ± 2.24**	47.18 ± 3.86**	44.61 ± 5.48**
IC_50_ (μM) (n_H_)	2.07 ± 0.21	—	16.31 ± 0.21**	6.44 ± 0.18**	10.50 ± 0.41**	—	—	8.29 ± 0.72**
	(1.87 ± 0.32)	—	(1.75 ± 0.11)	(1.55 ± 0.19)	(1.98 ± 0.07)	—	—	(1.34 ± 0.23)

Data are presented as mean ± SEM, and numbers of experiments (*n*) are shown in parenthesis. ^**^ *p* < 0.01 vs*.* WT.

### Model of HMQ1611 docking to hKv1.5 channel

**FIGURE 5:**
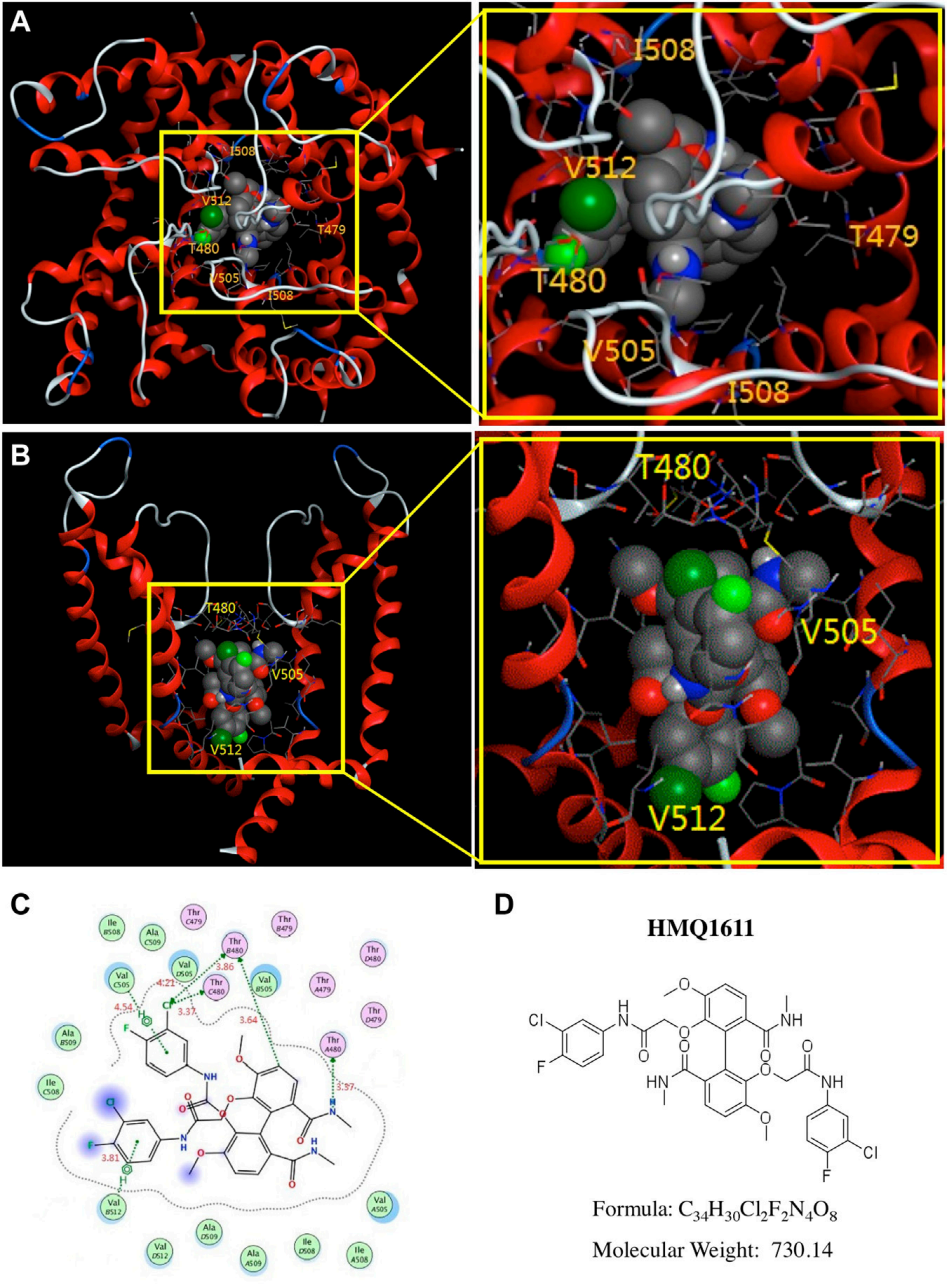
Molecular docking of HMQ1611 with Kv1.5 channel. **(A)** Top view of HMQ1611 docking to hKv1.5 channel pore, indicating the binding of HMQ1611 in central cavity of the channel. **(B)** A side view of HMQ1611 binding sites (T480, V505 and V512) in the S6 domain and selectivity filter of hKv1.5 channel pore. **(C)** Two-dimensional interaction map of HMQ1611 and hKv1.5 channel. The arrows indicate potential interactions between amino acid residues and HMQ1611. **(D)** The chemical structure of HMQ1611.

Docking simulations can provide molecular structural information of complexes between ion channels and ligands, which can be used to analyze corroborative data concerning the compound binding to different positions in the channel proteins. To investigate the binding modes of HMQ1611 within the pore cavity of hKv1.5 channel, a computational docking simulation of HMQ1611 with the hKv1.5 channel was conducted using a molecular docking software MOE 2016.0802. At the lowest free energy of binding model (-5.4 kcal/mol), we found that HMQ1611 is predicted to be located adjacent to the base of the ion selectivity filter of the hKv1.5 channel pore (Figures 5A,B), in proximity to T480 (at the base of ion selectivity filter) and, V505 and V512 (both located within S6 domain). Figure 5C shows the binding modes of HMQ1611 with the potential binding sites, in which V505 and V512 interacted with the compound via arene-H interactions and T480 interacted with the compound via CH-π interactions. The distances between HMQ1611 and the hydrogen atoms on T480, V505, and V512 were 3.37 Å, 3.64 Å, and 3.81 Å, respectively (Figure 5C).

## Discussion

The present study demonstrated that HMQ1611 inhibited the hKv1.5 current in a voltage-, concentration-, frequency-, and time-dependent manner with an average IC_50_ of 2.07 μM HMQ1611 preferentially interacted with the open state of the hKv1.5 channel, which took place within 5–10 s after exposure to the compound, reached steady-state inhibition within 3 min, and fully reversed within 10 min following removal of the compound (Figure 2D). The inhibitory effect of HMQ1611 on the hKv1.5 channel suggests that the derivative of symmetrical biphenyl can be a potential lead compound of a Kv1.5 channel blocker and is expected to be used as a new drug not only for the treatment of AF but also for cancer therapy. Mutational analyses indicated that amino acid residues located at the base of the selectivity filter (T479 and T480) and in the S6 segment (V505, I508, L510, V512, and V516) of the hKv1.5 channel appear to be important for the binding of HMQ1611 within the channel.

Induction and maintenance of AF are associated with shortening of the effective refractory period (ERP), and facilitating re-entry wavelets in the atria (Christophersen et al., 2013; Wijesurendra and Casadei, 2019). Therefore, the prolongation of myocardial refractoriness, primarily determined by APD, can exert antiarrhythmic actions against re-entrant based tachyarrhythmias. A major determinant of APD is the amount of repolarizing outward potassium currents, particularly those provided by three delayed rectifier potassium currents: *I*_Kur_, *I*_Kr_, and *I*_Ks_. However, as *I*_Kr_ and *I*_Ks_ are present in both the atrium and ventricle, blockade of these two currents can cause excessive prolongation of cardiac AP, which often increases the propensity for developing life-threatening ventricular arrhythmias (eg. TdP) and conversely increases susceptibility to AF through enhanced propensity for early after-depolarization (Christophersen et al., 2013; Chiamvimonvat et al., 2017; Peyronnet and Ravens, 2019; Nattel et al., 2020).

One method to circumvent the undesired side effects is to target ion channels which are predominantly expressed in the atria (e.g., *I*_Kur_), although their antifibrillatory efficacy is controversial (Voigt and Dobrev, 2016; Heijman et al., 2021). Therefore, extensive efforts have been made to develop pharmacological agents that modulate atrial-specific channels (EI-Haou et al., 2015; Peyronnet and Ravens, 2019; Geng et al., 2020; Borrego et al., 2021). In the present study, we found that HMQ1611 blocked the heterologous hKv1.5 channels with the following action characteristics that: 1) HMQ1611 exerted little effect on the initial peak current amplitudes but gradually reduced the current levels during the depolarizing steps; 2) HMQ1611 attenuated the hKv1.5 current during the depolarizing step in a concentration-dependent manner; 3) hKv1.5 current declined steeply at potentials ranging from -40 mV to 0 mV during exposure to HMQ1611, which corresponds to the voltage range of channel opening; 4) the K_D_ value derived by (*k*_-1_)/(*k*_+1_) was 4.48 μM, which is close to the value of IC_50_ (2.07 μM). The properties of HMQ1611 on the hKv1.5 channel suggest that this compound preferentially interacts with an open state of the channel. In addition, we found that the deactivation time constant of hKv1.5 was not influenced by HMQ1611 and the inhibitory effects of HMQ1611 on the channel were reversible upon washout (Figure 2D), suggesting that the speed at which the compound dissociated from the Kv1.5 channel was not affected, and the channel regained its intrinsic biophysical properties and normal function without any delay. As a result, the “crossover” phenomenon in the tail currents caused by HMQ1611 was not observed in the hKv1.5 channel (Kojima et al., 2015; Lee et al., 2016; Fukushima et al., 2020). In the present study, intracellular dialysis of cells with 5 μM HMQ1611 for 4 min after the formation of the whole-cell configuration did not change the Kv1.5 currents recorded, implying that the inhibition of this compound on the channel occurred from the external side of the cell membrane.

HMQ1611 is a taspine analogue with a symmetrical biphenyl (Figure 5D), which has been shown to have potent anticancer activity primarily by blocking receptor tyrosine kinase (Lu et al., 2012; Zhan et al., 2012). LY294002 (a compound derived from the flavonoid quercetin), with the same biphenyl scaffold, is also a potent anticancer agent. However, despite sharing the same biphenyl structure, the antineoplastic effect of LY294002 is mediated by inhibition of PI3K-dependent Akt phosphorylation and kinase activity (Ebrahimi et al., 2017), rather than receptor tyrosine kinase (Vlahos et al., 1994). Recently, increasing evidence suggests that Kv1.5 is overexpressed in many types of human carcinomas, including stomach, pancreatic, colon, brain, bladder, skin, glioma, lymphoma, and lung cancers (Felipe et al., 2012; Aung et al., 2019; Serrano-Novillo et al., 2019). Although the Kv1.5 expression in some healthy tissues including brain is low, the expression is increased in most tumors (Bielanska et al., 2009). Kv1.5 participates in neoplastic processes, such as proliferation, migration, and invasion (Villalonga et al., 2008; Serrano-Novillo et al., 2019). Silencing Kv1.5 expression in osteosarcoma cells inhibits cell proliferation and induces cell cycle arrest as well as cell apoptosis (Wu et al., 2015). Therefore, it is proposed that Kv1.5 contributes to cancer development, and the regulation of this channel would be useful as a pharmacological tool in anticancer therapies. Both HMQ1611 and LY294002 show potent Kv1.5 channel blocking effect, it can thus be conceivably hypothesized that, in addition to inhibiting tyrosine kinase or PI3K, the inhibition of growth of some human carcinomas by the compounds is also involved with the blockade of hKv1.5. Unfortunately, the inhibition of tyrosine kinase or PI3K could inevitably result in some side effects for the treatment of AF patients. Further investigations are warranted to evaluate whether and how the antineoplastic actions of HMQ1611 involve in Kv1.5 blocking.

Previous mutational analyses using site-directed mutagenesis have revealed that amino acid residues in the Kv1.5 channel, including T462, H463, T479, T480, R487, A501, I502, V505, I508, A509, L510, V512, and V516, could be potential binding sites for various Kv1.5 channel blockers (Eldstrom et al., 2007; Wu et al., 2009; Bai et al., 2015; Fukushima et al., 2020). Among these amino acid residues in the Kv1.5 channel, T462 and H463 are located at the outer mouth of the pore helix; T479, T480, V505, I508, A509, V512, P513, and V516 are positioned facing the inner cavity of the channel pore, while I502 and L510 are pointed away from the central cavity of the pore (Decher et al., 2006; Eldstrom et al., 2007; Tikhonov and Zhorov, 2014; Fukushima et al., 2020). In the present study, we found that the hKv1.5 mutants, including T479A, T480A, V505A, I508A, L510A, V512A, and V516A, significantly reduced the inhibitory potency of HMQ1611 on the hKv1.5 WT channel (Figures 4A,B). Amino acid residues T479A, T480A, V505A, I508A, V512A, and V516A can directly interact with the compound in the hKv1.5 channel and affect channel function due to their positions in the inner cavity of the hKv1.5 channel. In contrast, L510 pointed away from the central cavity of the pore. A possible explanation for the reduction in sensitivity of the L510A channel to HMQ1611 is that of allosteric conformational change, which leads to the amino acid residues undergoing rotation and exposing L510 to the compound (Eldstrom et al., 2007; Bai et al., 2015; Fukushima et al., 2020). A similar phenomenon was observed for other compounds such as AVE0118 and LY294002 (Decher et al., 2006; Wu et al., 2009). The data in Figure 4 suggest that the base of the selectivity filter in the deep pore (T479 and T480) and the S6 domain that lies deeper than the selectivity filter (five residues from V505 to V516) are the areas where HMQ1611 could bind to the hKv1.5 channel.

Molecular docking is an important auxiliary tool for predicting the predominant binding modes of a compound with an ion channel protein, particularly the binding position of the compound within the channel and the interaction energy between the compound and the residue helix. The docking simulation data of the present study showed that residues T480, V505, and V512 are the functional molecular docking sites of HMQ1611 in the Kv1.5 channel (Figure 5C). One hydrogen bond was generated between amino group and T480 with length of 3.64 Å. Two phenyl rings of terminal 3-chloro-4-fluoroaniline formed tow π-π interactions with phenyl ring of V505 and V512 in the S6 segment with distance of 4.54 Å and 3.81 Å, respectively. π-π interaction is a particular type of dispersion force from van der Waals forces, which is established between unsaturated (poly)cyclic molecules. There are two additional phenyl rings attached on the biphenyl scaffold of HMQ1611. Therefore, compared with those of LY294002, more interactions were observed in HMQ1611, including two π-π interactions with V505 and V512. Taken together, our findings reveal the importance of these residues in docking of HMQ1611 to the Kv1.5 pore homology model and partially corroborate the binding sites identified using a site-directed mutagenesis approach. Decher et al. (2006) compared the docking modes of S0100176 and AVE0118 in the Kv1.5 homology model. They proposed that, contrary to AVE0118, the compactly shaped S0100176 docked within the pore of the Kv1.5 channel homology model in such a configuration that the binding side-chain of residues extended from T480 to V512, which did not slow the rate of Kv1.5 current deactivation. Similarly, the compact shape of HMQ1611 (Figure 5D) docked to the inner pore residues T480, V505, and V512 in the Kv1.5 channel homology model and did not cause crossover of the tail current traces in the absence and presence of HMQ1611. The docking profile may provide a hint to the study or rational design of a new Kv1.5 blocker.

In conclusion, the present study found that HMQ1611 preferentially inhibits the hKv1.5 current in the open state with an average IC_50_ of 2.07 μM. Seven residues including T480, V505, and V512 are putative binding sites for HMQ1611 in central cavity of hKv1.5 channel. These findings are of great theoretical significance and practical value for the research and development of new Kv1.5 blockers for the treatment of Kv1.5 channel-associated diseases, such as AF and cancer.

## Data Availability

The raw data supporting the conclusions of this article will be made available by the authors, without undue reservation.
